# Quality assurance of VMAT on flattened and flattening filter‐free accelerators using a high spatial resolution detector

**DOI:** 10.1002/acm2.12864

**Published:** 2020-04-11

**Authors:** F. S. Matar, D. Wilkinson, J. Davis, G. Biasi, T. Causer, I. Fuduli, O. Brace, N. Stansook, M. Carolan, A. B. Rosenfeld, Marco Petasecca

**Affiliations:** ^1^ Centre for Medical Radiation Physics University of Wollongong Wollongong Australia; ^2^ Illawarra Cancer Care Centre Wollongong Hospital Wollongong Australia; ^3^ Illawarra Health and Medical Research Institute – IHMRI Wollongong Australia; ^4^ Department of Radiology Faculty of Medicine Mahidol University Bangkok Thailand

**Keywords:** FF, FFF, MLC, QA, solid‐state detectors, VMAT

## Abstract

**Purpose:**

This study investigated the use of high spatial resolution solid‐state detectors (DUO and Octa) combined with an inclinometer for machine‐based quality assurance (QA) of Volumetric Modulated Arc Therapy (VMAT) with flattened and flattening filter‐free beams.

**Method:**

The proposed system was inserted in the accessory tray of the gantry head of a Varian 21iX Clinac and a Truebeam linear accelerator. Mutual dependence of the dose rate (DR) and gantry speed (GS) was assessed using the standard Varian customer acceptance plan (CAP). The multi‐leaf collimator (MLC) leaf speed was evaluated under static gantry conditions in directions parallel and orthogonal to gravity as well as under dynamic gantry conditions. Measurements were compared to machine log files.

**Results:**

DR and GS as a function of gantry angle were reconstructed using the DUO/inclinometer and in agreement to within 1% with the machine log files in the sectors of constant DR and GS. The MLC leaf speeds agreed with the nominal speeds and those extracted from the machine log files to within 0.03 cm s^−1^. The effect of gravity on the leaf motion was only observed when the leaves traveled faster than the nominal maximum velocity stated by the vendor. Under dynamic gantry conditions, MLC leaf speeds ranging between 0.33 and 1.42 cm s^−1^ were evaluated. Comparing the average MLC leaf speeds with the machine log files found differences between 0.9% and 5.7%, with the largest discrepancy occurring under conditions of fastest leaf velocity, lowest DR and lowest detector signal.

**Conclusions:**

The investigation on the use of solid‐state detectors in combination with an inclinometer has demonstrated the capability to provide efficient and independent verification of DR, GS, and MLC leaf speed during dynamic VMAT delivery. Good agreement with machine log files suggests the detector/inclinometer system is a useful tool for machine‐specific VMAT QA.

## 1 Introduction

Volumetric Modulated Arc Therapy (VMAT) is an extension of Intensity Modulated Radiation Therapy (IMRT). VMAT delivery is accomplished by simultaneous modulation of the dose rate (DR), gantry speed (GS) and Multi‐leaf collimator (MLC) apertures^1^ offering the same clinical advantages as IMRT, namely high conformal dose to the target and organ at risk sparing, but with less monitor units (MU) and a shorter delivery time.^2^, ^3^, ^4^ VMAT's advantages can be realized if a comprehensive commissioning and quality assurance (QA) program are routinely implemented.^5^ Previous studies^6^, ^7^ have proposed a set of tests that are specific to the implementation of Varian RapidArc (Varian Medical Systems, Palo Alto, CA, USA). However, due to difficulties related to film dosimetry and portal images, the delivery aspects of VMAT could not be assessed separately and with sufficient gantry‐angle resolution. The NCS Code of Practice (CoP) Report 24^8^ published in 2015 outlines guidelines for the commissioning and QA of VMAT using multiple machine‐independent dosimetry systems. The CoP suggests that understanding the dynamic behavior of VMAT is best achieved by assessing the linac's dynamic components both individually and collectively and as a function of gantry angle. Following the recommendations of the NCS CoP Report 24, Barnes et al.^9^ examined the coordination between GS and relative dose profiles using a gantry‐mounted ionization chamber array (MatriXX, IBA Dosimetry, Germany) in conjunction with an inclinometer. The limited spatial resolution of the array detector though, maybe the reason MLC leaf speed verification was not included in the study. Further, the measured parameters were not evaluated as a function of gantry angle. Electronic portal imaging devices (EPIDs) have become a standard component in the current linac designs.^10^, ^11^ Commercial amorphous‐silicon EPIDs have a pixel size of 0.392 mm^2^ × 0.392 mm^2^ and spatial resolution of 0.784 mm.^12^, ^13^ Recently, an EPID‐ based system^14^ has been tested for VMAT QA. This system showed good agreement with a plan for all dynamic parameters including MLC motion, however, the readout of gantry angle information extracted from the On‐Board Imaging system may not be considered as machine‐independent. In addition, it has been reported that EPIDs have a non‐linear response to low MU^15^, ^16^ in IMRT and VMAT deliveries and have shown discrepancies with respect to the expected dose in small MU fields.^17^ An alternative method for VMAT QA is the use of machine log files.^18^, ^19^, ^20^ During dynamic deliveries, the log files record cumulative MU index and positional information (gantry and MLCs) from machine encoders.^21^ This information requires prior validation with an external device and is only retrieved once delivery is completed. Therefore, machine log files do not offer real‐time linac‐independent analysis. In this work, we report on the use of a high spatial resolution (0.2 mm) solid‐state detector (DUO), operated in transmission mode which records the current generated in each pixel by the flux of photons and combined to an inclinometer to provide an independent measurement of the angular position of the gantry. The two datasets are synchronized with the sync pulse of the linac allowing for highly detailed timing information available to perform independent machine‐specific QA for VMAT based on the CoP. The system is capable of assessing the DR and the GS as a function of gantry angle in both flattened and unflattened beams. Dynamic MLC movement is a major component of VMAT delivery. In fact, MLC leaf speed has been proven to have a greater impact on the accuracy of VMAT delivery in comparison to DR and GS,^22^, ^23^ and was found to be the main contributor to inaccuracies in MLC positioning.^6^, ^18^, ^24^ Therefore, the DUO was replaced by another high spatial resolution array detector named Octa that allowed simultaneous speed assessment of multiple MLC leaves during arc deliveries. Both the DUO and Octa have a sensitive volume of 0.032 mm^2^ which is substantially smaller than that of EPIDs. The DUO and Octa's sub‐millimeter spatial resolution is essential in the precise evaluation of MLC‐defined fields that are commonly used in VMAT plans while the millisecond time resolution allows finite and time‐resolved evaluation of the DR profiles and GS. That in combination with the easy setup and simple calibration procedure provides a device with the capability to simplify the application of the CoP and its use in clinical practice over existing commercial designs.^25^, ^26^, ^27^

## 2 Materials and Methods

All measurements were performed using a 6 MV flattening filter beam on a Varian 21iX and a 10 MV flattening filter‐free beam on a Varian Truebeam. Each linac was equipped with a Millennium 120‐leaf MLC organized in two banks (A and B), each with 60 round‐end leaves (Varian Medical Systems, Palo Alto, CA, USA). The 40 central leaves in each bank are 5 mm in width projected at isocenter, while the outer 20 leaves are 10 mm. Varian machines create dynamic log files that record gantry angle, MLC positions and cumulative MU information during each dynamic delivery at 50 and 20 ms intervals for the 21iX and Truebeam, respectively. Varian machine log files were used as a point of comparison for this study.

### 2.1 Proposed QA instrumentation

**Fig. 1:**
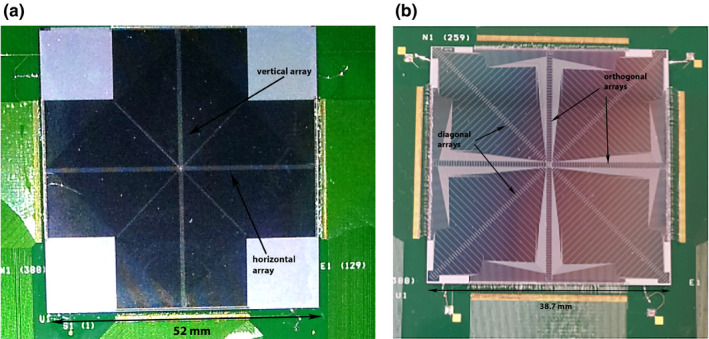
(a) The DUO detector featuring the two orthogonal linear arrays. (b) The Octa detector with its 4 linear arrays.

The DUO [Fig. 1(a)] is a monolithic silicon detector, consisting of 505 sensitive volumes arranged in two orthogonal linear arrays. Each volume has an area of 0.04 mm^2^ × 0.8 mm^2^ and the five central volumes intersecting the arrays are 0.18 mm^2^ × 0.18 mm^2^ in size. The sensitive volumes are equally spaced with a center‐to‐center distance of 0.2 mm giving the detector overall dimensions of 52 mm^2^ × 52 mm^2^. The DUO has been characterized for machine‐specific QA in small radiation fields produced by megavoltage‐flattened beams during in‐phantom studies.^28^, ^29^

To allow the speed evaluation of multiple MLC leaves, the DUO detector was replaced by another solid‐state monolithic sensor named Octa.^30^, ^31^ The Octa displayed in Fig. 1(b), consists of additional two linear micro‐strip arrays aligned diagonally (or 45°) to the vertical and horizontal arrays comprising of 129 sensitive volumes with a center‐to‐center distance of 0.30 mm in the orthogonal and 0.43 mm in the diagonal arrays. Each sensitive volume has an area of 0.04 mm^2^ × 0.8 mm^2^ excluding the nine volumes intersecting the arrays which are 0.16 mm^2^ × 0.2 mm^2^ in size. The 512 sensitive volumes in the Octa sensor cover an overall detector area of 38.7 mm^2^ × 38.7 mm^2^.

**Fig. 2:**
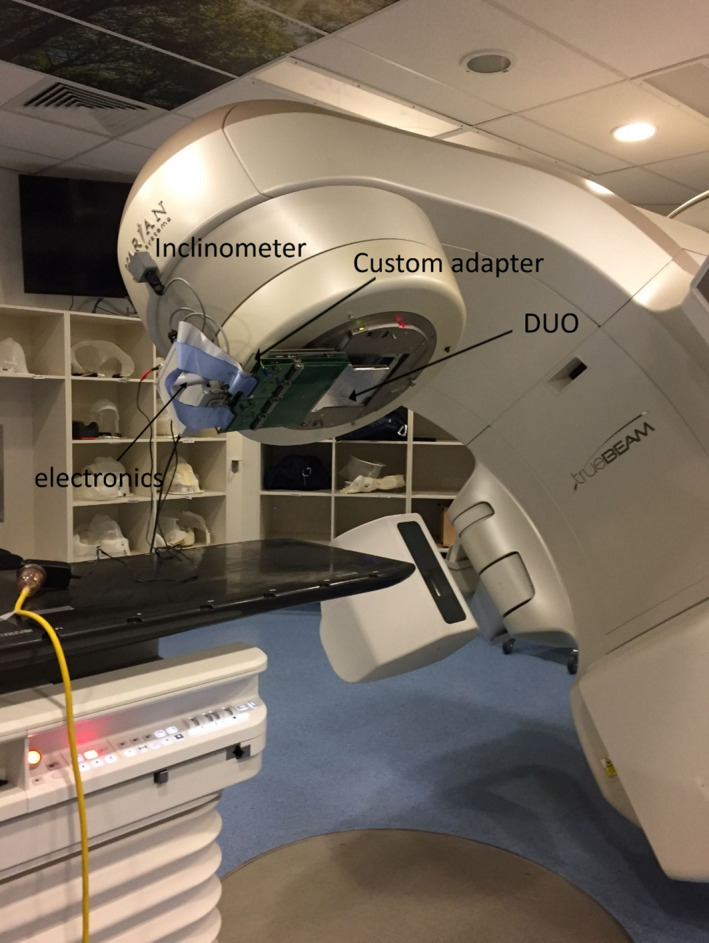
Experimental setup: The DUO was attached to a Varian accessory tray and inserted into the accessory tray slot. The inclinometer was mounted on the linac head.

The detector was stacked between two 5 mm thick PMMA slabs with a recess in the slab on top of its active area and covered with an aluminum film to shield it from external light and electromagnetic noise. The detector was synchronized to a digital inclinometer by a fast data acquisition system^32^ based on a Field Programmable Gate Array. The inclinometer was an ADIS16209 from Texas Instruments (TI – Nexville US) with a bi‐directional accuracy of 0.1° and a resolution of 0.025°.^33^ The inclinometer was attached to the linac head and calibrated against the linac gantry position encoder at 0° (IEC scale). The detector assembly was fixed to a custom mechanical adapter attached to a Varian accessory tray and lodged into the designated tray slot at a source‐to‐detector distance of approximately 60 cm (Fig. 2). This setup placed the central sensitive volumes of the detector perpendicular to the incident radiation beam during gantry rotation to eliminate any angular dependence of the detector's response. The vertical array was aligned with respect to the linac central axis using the smallest available rectangular radiation fields and Vernier micro‐positioners that shift the detector with respect to the beam in the superior‐inferior and lateral directions.

### 2.2 Detector calibration

The linearity of the DUO's response to irradiations that ranged from 2 to 1000 MU was investigated at fixed DRs of 300, 400 and 600 MU min^−1^ delivered on the 21iX with 6 MV flattened beams and 1200 MU min^−1^ delivered on the Truebeam with 10 MV unflattened beams and an MLC‐defined field size of 10 cm^2^ × 10 cm^2^ at isocenter. The calibration factors that convert the charge collected by the detector to delivered MU were obtained from the slope of the linear fit.

### 2.3 Mutual dependence of dose rate and gantry speed

The mutual dependence of DR and GS during VMAT deliveries was investigated with the use of the Customer Acceptance Plan (CAP). The CAP test is a standard plan provided by Varian at acceptance across all centers and has been reported in the literature.^9^ The plan is designed to demonstrate the linac's dynamic performance through the full range of motions within the manufacturer's specifications. The transitions in the GS, DR and MLC leaf speed (when applicable) contain various clinically possible transitions that can occur in VMAT plans. In order to independently measure the DR and GS with the DUO, the plan was customized to produce a static MLC aperture of 1 cm^2^ × 10 cm^2^ centerd on one axis of the array detector. The jaws were set to 10 cm^2^ × 10 cm^2^. The plan comprised of different dose sectors, each with a different MU weighting requiring a particular combination of DR and GS. These combinations included:

DRs: 599, 499, 35 and 0 MU min^−1^ corresponding to GSs: 0.50, 1.00 and 5.00° s^−1^ for the 21iX deliveries.

DRs: 799, 593, 42 and 0 MU min^−1^ corresponding to GSs: 0.66, 1.33 and 6.00° s^−1^ for the Truebeam deliveries. The maximum DR was set to 1200 MU min^−1^ on the Truebeam as an upper limit because the electronics would have been saturated by the flux of photons at higher DRs. The detector itself has virtually no DR limit within the DR range of modern linacs.

The same plan was delivered in both the clockwise and counter‐clockwise rotational directions between gantry angles of −128˚ and 128˚ to investigate any directional dependence of the dynamic delivery.

### 2.4 Dose rate and gantry speed optimization

To reduce the noise associated with the instantaneous fluctuations in the DR and GS, an integrated signal was calculated over 90 and 14 frames corresponding to time intervals of 250 and 100 ms for the 21iX and Truebeam, respectively. The GS was calculated as the difference in the acquired gantry positions over the same time intervals. For comparison, the same time intervals were used to average the machine log file data (ie. an average of 5 log file entries).

### 2.5 MLC leaf speed

**Fig. 3:**
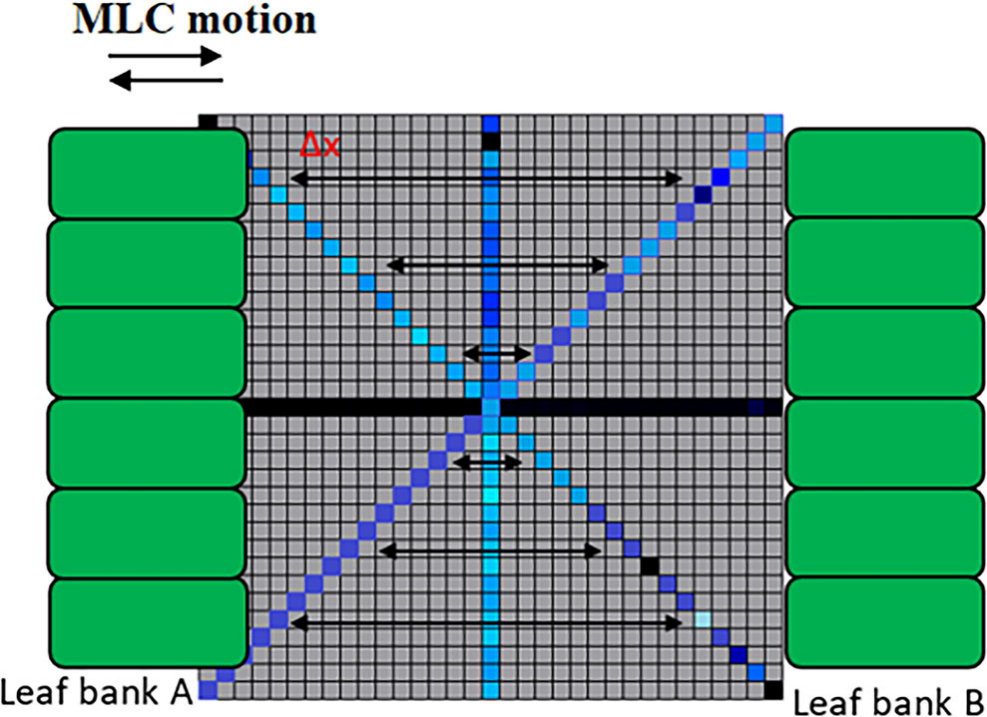
A diagram of the Octa detector arrays and the multi‐leaf collimator (MLC) leaves' motion with respect to the detector arrays (not to scale).

The Octa detector has two linear arrays oriented at ±45° with respect to the superior‐inferior axis of the MLCs. This geometrical arrangement can be exploited to provide simultaneous speed evaluation of multiple leaves. Figure 3 shows schematics of the MLC banks and the MLC motion with respect to the Octa detector. While the MLC leaves traverse the area of the detector, the speed was measured by analyzing the intensity profiles of the sensitive volumes located on the adjacent diagonal arrays as they respond to irradiation under the open aperture. Leaf speed was calculated as the leaf displacement divided by displacement time. Leaf displacement was determined by the distance the leaves travel across the area of the detector calculated based on the known geometry of the detector. In order to evaluate the distance covered by the leaves, a correction factor was used to correct for the projection of the leaves on the detector plane with respect to isocenter.

**Fig. 4:**
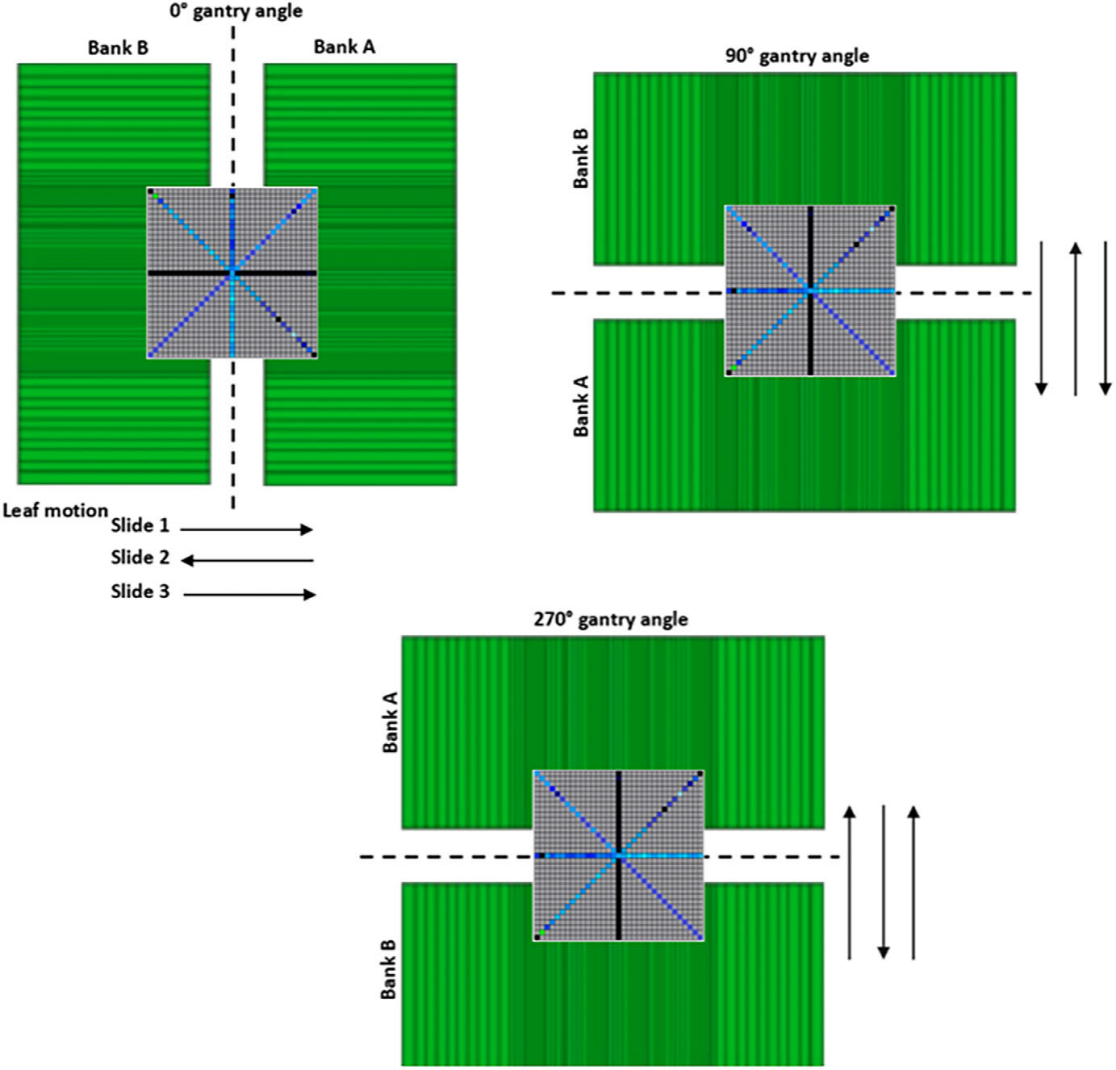
A schematic of the multi‐leaf collimator (MLC) leaves' motion with respect to the Octa detector arrays in the MLC leaf tests under static gantry conditions at gantry positions of 0°, 90°, and 270°.

The leaf speed was verified under static and dynamic gantry conditions with the use of a fixed 2‐cm MLC slit that sweeps across the array detector. Under static gantry conditions, tests with two different nominal speeds of 1.87 and 2.80 cm s^−1^ were delivered. The latter chosen to be above the clinical limit of 2.50 cm s^−1^ in order to identify any limitations in leaf performance. In each test, the MLC banks perform 3 translations across the linac central axis at gantry positions of 0°, 90°, and 270° in order to examine the influence of gravity on the leaf motion. Figure 4 represents schematics of the position of the detector with respect to the MLC leaf banks and the MLC leaf movement at the three selected gantry positions.

Under dynamic gantry conditions, the CAP test described in Section 2.C was modified to incorporate MLC leaf motion at varying leaf speeds between control points with simultaneous modulation of DR and GS. This test was delivered on the Truebeam with the 10 MV flattening filter‐free beam. Measurements with the Octa device were compared to the machine log files.

## 3 Results

### 3.1 Detector calibration

The DUO detector exhibited a linear response with an accumulated dose that ranged from 2 to 1000 MU with regression coefficients of 0.99 and 1 for the 21iX and the Truebeam deliveries, respectively. The reproducibility of the detector's response under different DRs was within ±0.5% for irradiations greater than 5 MU and ±1.6% for irradiations of 5 MU and lower. The slopes of the linear fit were used to relate the charge collected by the detector to MU delivered. The detector's response was corrected for field size dependence due to reduced scatter when smaller radiation fields were used. The respective calibration factors for the 21iX and the Truebeam were 11.90 ± 0.04 and 1.88 ± 0.01 nC MU^−1^. The difference in the calibration factors is related to the variation in the DR modulation techniques between the two linacs. On the 21iX, the DR is modulated using a pulse dropping technique while the sync pulse and the electron trigger gun conserve the same time‐based frequency (360 Hz). Therefore, a static phase lock loop (PLL) was adopted in order to synchronize the data acquisition to the linac. On the Truebeam, the DR is adjusted by both pulse‐dropping and continuous modulation of the time‐based frequency of the synch pulse with frequencies that vary from 100 up to 360 Hz. To ensure synchronization between the data acquisition system and the Truebeam, a dynamic PLL was designed. This enables the triggering of the acquisition of the charge generated in the detector to each trigger pulse. For this technique to succeed, the integration time with the dynamic PLL must be shorter than that with a static PLL. Therefore, the charge collected from the same flux of photons is significantly smaller on the Truebeam than on the 21iX since it is integrated over a shorter time. Fuduli et al.^32^ provide a detailed description of the data acquisition system.

### 3.2 Mutual dependence of dose rate and gantry speed

**Fig. 5:**
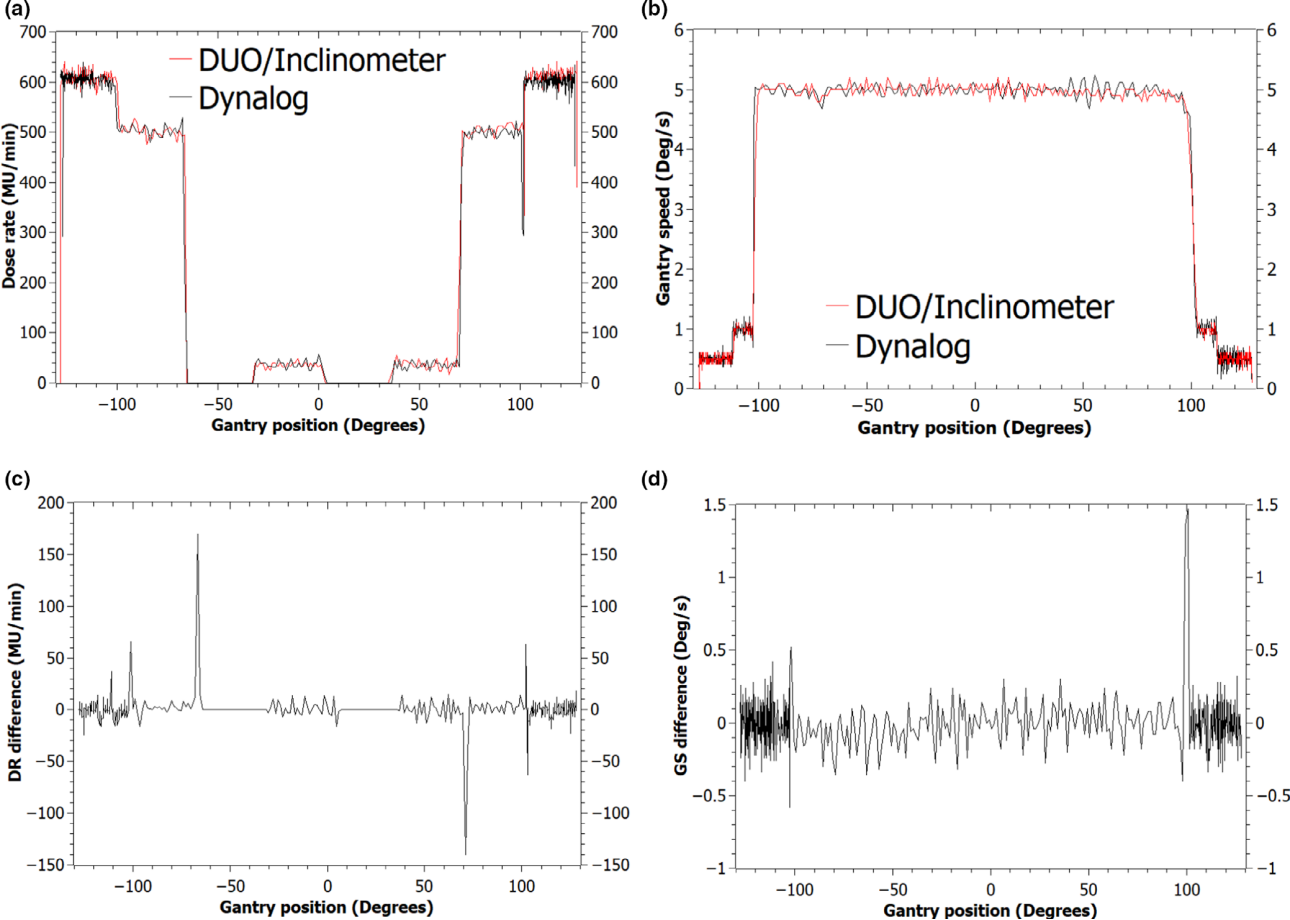
Dose rate versus gantry angle (a) and Gantry speed versus gantry angle (b) measured with the DUO/inclinometer and compared to the dynalog files data. The difference in the dose rate (c) and gantry speed (d) between the DUO/inclinometer and the dynalog files plotted as a function of gantry angle.

**Fig. 6:**
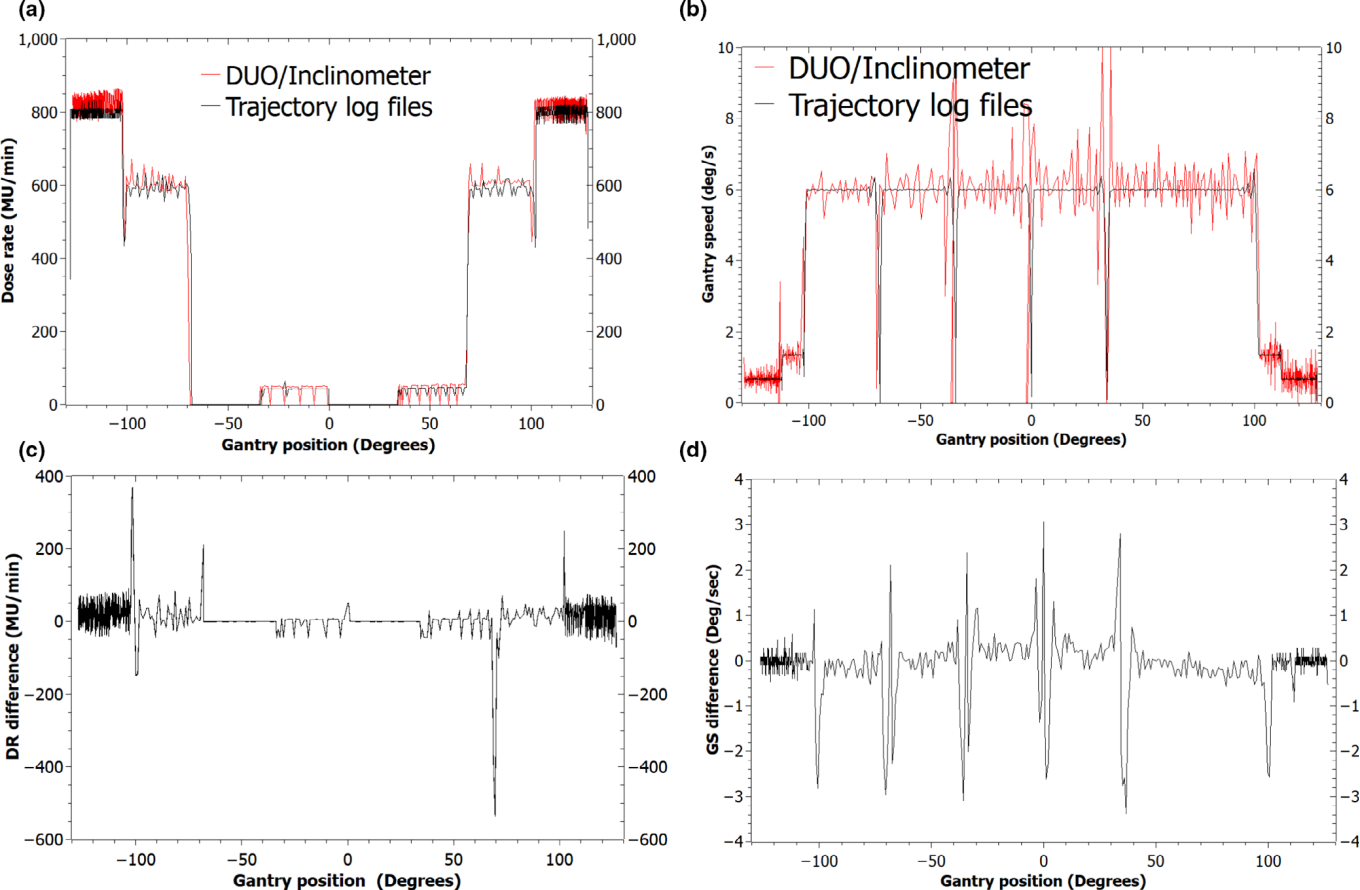
Dose rate (a) and Gantry speed (b) measured with the DUO/inclinometer compared to the trajectory log files and plotted against gantry angle. The difference in the dose rate (c) and gantry speed (d) between the DUO/inclinometer and the trajectory log files plotted as a function of gantry angle.

The agreement between the detector system and the dynamic log files (dynalog files) was found to be independent of gantry direction and as such only the counter‐clockwise arc results are presented. The DR and GS measured with the DUO/inclinometer and compared to the machine log files are shown in Figs. 5 and 6 for the 21iX and the Truebeam deliveries, respectively.

#### 3.2.1 Varian Clinac 21iX

The plan delivers four distinct DR and GS combination sectors during the arc delivery and these are repeated on either side of the 0° gantry position (Fig. 5). The DUO measured all four combinations as well as changes in DR and GS between control points.

The average of the parameters measured with the DUO/inclinometer and recorded with the dynalog files agreed to within 1% in the sectors with constant DR and GS [Figs. 5(a) and 5(b)]. During the transition between control points, differences in the DR measured with the DUO and recorded with the dynalog files varied between ±20 and ±150 MU min^−1^ [Fig. 5(c)]. The maximum difference occurred at the control points with the largest change in DR from 0 to 499 and 499 to 0 MU min^−1^. The difference in the GS seen in Fig. 5(d) between the dynalog data and the inclinometer measurements varied between ±0.22° s^−1^ with a maximum difference of 1.50° s^−1^ at the control points of largest GS acceleration/ deceleration from 1.00° to 5.00° and 5.00° to 1.00° s^−1^.

#### 3.2.2 Varian Truebeam

Figure 6 shows the reconstructed DR and GS as a function of gantry angle in the counter‐clockwise arc measured with the DUO/inclinometer for the 10 MV flattening filter‐free beam and compared to the trajectory log files. The DUO/inclinometer registered high‐frequency fluctuations in both the DR and GS traces.

The DUO/inclinometer and the trajectory log files agreed to within 1% in the average DR. As observed in Fig. 6(a), both systems detected a drop below the expected DR at the gantry position of ±102°. These deviations were coincident with the transitions in the GS during maximum acceleration and deceleration. Other discrepancies in the reconstructed DR between the DUO/inclinometer and the trajectory log files were observed at the gantry positions of ±68˚ where the DR transitions from 0 to 593 MU min^−1^ and vice versa [Fig. 6(c)].

The GS on the Truebeam measured with the inclinometer revealed larger fluctuations than those observed on the 21iX, but with values that constantly ranged around the average nominal velocity. Another notable feature was the behavior of the gantry movement which deviated from the planned continuous motion. This deviation was detected in both the DUO/inclinometer as well as in the trajectory log files data. The gantry was found to shortly pause at the transition between control points as observed in Fig. 6(b) at gantry positions of ±34°, 0°, and −68°. The speed measured with the inclinometer in and around the time of these pauses showed a speed that exceeded the maximum GS of 6.00° s^−1^ with the inclinometer measuring instantaneous speeds of up to 9.97° s^−1^. These speeds were not reflected in the trajectory log files, are likely erroneous and can be attributed to the response of the inclinometer under strong vibrations generated in the linac gantry head during moments of extreme accelerations and decelerations of the gantry. The effect of vibrations on the response of the inclinometer can be reduced using a low‐pass filter. The inclinometer provides a few tools to achieve this, however, the command settings required to activate these functions have not been used and would entail further development of the electronics' readout.

### 3.3 MLC leaf speed

The MLC leaf speed was assessed under the static gantry on the 21iX and the Truebeam. The MLC leaf speed obtained with the dynalog files was 1.89 ± 0.20 cm s^−1^ while the trajectory log files recorded an average of 1.87 ± 0.02 cm s^−1^. In comparison, the Octa measured an average speed of 1.89 ± 0.03 cm s^−1^ on both machines. At this speed, the MLC motion is independent of the gantry angle as no discernible differences between the three translations at the gantry positions of 0°, 90°, and 270° were observed. This demonstrates that the MLC motion at the selected speed was unaffected by the force of gravity.

In the maximum leaf speed test on the 21iX, at the 0° gantry position where the leaves move orthogonal to the force of gravity, the Octa and the dynalog files reported an average of 2.86 ± 0.03 cm s^−1^ and 2.81 ± 0.24 cm s^−1^ respectively. At the two gantry angular positions of 90° and 270°, in one translation, the detector measured a dramatic reduction in the speed (approximately −33%), whereas the dynalog files reported a speed that oscillated between 0 and 7.99 cm s^−1^. Conversely, on the Truebeam, an average leaf speed of 2.50 ± 0.02 cm s^−1^ was retrieved from the trajectory log files, while the Octa device measured an average of 2.52 ± 0.03 cm s^−1^. The MLC leaf speed was found to be independent of gantry angle.

The average MLC leaf speeds measured using the Octa detector under dynamic gantry conditions delivered with simultaneous modulation of DR and GS at varying leaf speeds are presented in Table 1.

**Table 1:** Multi‐leaf collimator (MLC) leaf speeds under dynamic gantry conditions with simultaneous modulation of dose rate and gantry speed measured with the Octa and compared to the trajectory log files.

Nominal DRs (MU min^−1^)	799	799	593	0	42
Octa (cm s^−1^)	0.33 ± 0.01	1.09 ± 0.02	1.42 ± 0.14	—	1.49 ± 0.37
Trajectory log files (cm s^−1^)	0.33 ± 0.02	1.07 ± 0.05	1.42 ± 0.12	1.42 ± 0.02	1.42 ± 0.02
% difference	0.9	1.9	2.1	—	5.7

Deviations in the speed were calculated as percentage differences between the Octa and the trajectory log files data. The difference varied from 0.9% to 5.7% with the maximum discrepancy measured at the speed of 1.42 cm s^−1^ and the DR of 42 MU min^−1^ while the minimum was obtained at the speed of 0.33 cm s^−1^ and the DR of 799 MU min^−1^. Since the proposed method was based on analyzing the intensity profiles of the sensitive volumes' signal, the speed was not verified at the angular sectors of 0 DR. Measurement uncertainty when determining the MLC leaves' position was found to be ±0.32 mm.

## 4 Discussion

The purpose of this study was to investigate the use of high spatial resolution solid‐state detectors combined to an inclinometer as a machine‐specific QA device for VMAT. VMAT plans were designed based on the recommendations of the NCS Code of Practice Report 24^8^ to provide simultaneous information relating to the dynamic relationship between DR, GS, and MLC during arc delivery. The DUO/inclinometer measured the DR and GS in the CAP test deliveries on the 21iX and the Truebeam in flattened and unflattened beams (Figs. 5 and 6). Through comparison with the machine log files, agreement to within 1% of the average measured quantities was observed in the sections of the arc where the DR and GS were constant.

The deviations in the DR from the expected values in the CAP test were captured by the DUO/inclinometer system and were also evident in the trajectory log files. At these points in the arc (±102°) the gantry does not reach the planned speed instantly and hence the delivery system reduces the DR to allow for GS adjustment. These deviations are indications of the mutual dependence between the two parameters to achieve the desired dose delivery during VMAT.

Variations in the recorded DRs between the proposed system and the machine log files tended to be at the transition between control points. In Figs. 5 and 6, these variations appear as gantry positional differences (x‐axis) but are attributed to a synchronization issue between the machine log files and the inclinometer data. The inclinometer trails behind the machine log files at the transition between slow and maximum GS by a temporal maximum period of 1 s and 600 ms on the 21iX and the Truebeam respectively. Agreement between the two systems was returned once the gantry reached a constant velocity. The tests showed that there was no lag between the DUO and the inclinometer readings.

The fluctuations observed in the measured DR and GS on the 21iX and the Truebeam (Figs. 5 and 6) are a reflection of the feedback control mechanism between the linac and the MU control systems. The DR and GS measurements exhibited larger fluctuations on the Truebeam than the 21iX. This is related to the mounting of the inclinometer and the number of data points that have been used to calculate each parameter. The inclinometer was placed on the gantry of the 21iX, however, due to the curved surface of the Truebeam gantry, the inclinometer was alternatively mounted on the linac head. This position has the drawback to induce a larger amount of lateral vibrations in the sensor, which consequently produced a noisier dataset. Additionally, each value of the DR and the GS measured on the Truebeam deliveries was obtained over 14 data points as opposed to 90 data points on the 21iX.

Comparing the results of the CAP tests on the two treatment machines, some unexpected behavior in the gantry motion was observed on the Truebeam whereby the gantry momentarily paused at specific control points [Fig. 6(b)]. This was not noticed on the 21iX, however, in both cases, the behavior of the linac was accurately recorded by both the DUO/inclinometer and the trajectory log files. The reason for the difference in behavior between the two treatment machines, in what was intended to be the same test, may be explained by the priority setting of the leading parameters. The leading parameters are the gantry angle and the delivered MU for the 21iX and the Truebeam, respectively. All dynamic parameters are synchronized to that leading parameter and any deviations in the latter will cause deviations in the subjugated parameters. The Truebeam system will, therefore, monitor its dynamic components (GS and MLC motion) as a function of the leading parameter (delivered MU) and if a deviation from the plan is observed, the linac will either correct the GS or hold delivery depending on the deviation from the tolerance level.^8^

Under static gantry conditions, the MLC leaf speeds measured with the Octa were found to agree with the nominal leaf speeds and the machine log files to within 0.03 cm s^−1^. The influence of gravity on the accuracy of the leaf speed was assessed by delivering the sweeping window tests at gantry positions of 90° and 270°. At these positions, when the MLC leaves traveled at a velocity that exceeded the nominated performance limit, the MLC motion was significantly affected by gravity. This result is consistent with the results previously reported in the literature.^19^ Examining the dynalog files for these maximum leaf speeds, the MLC motion appeared to oscillate between instantaneous speeds of 0 and 7.99 cm s^−1^. This anomaly may be related to the latency in the feedback loop between the linac and the MLC control systems. The linac instructs the MLC motors to drive the MLC leaves depending on their actual position with respect to the instructed one. This may cause the MLC leaves to accelerate or decelerate when found, by the linac encoder, to trail behind or surpass their intended position, respectively. Nevertheless, the recorded speed of 7.99 cm s^−1^, at these extreme delivery conditions, may not manifest the actual MLC leaf speed as the dynalog files have been shown to report erroneous values.^34^

On the Truebeam, the linac exhibited a better compliance with the maximum speed limit. Instead of attempting to achieve the nominal leaf motion of 2.80 cm s^−1^, the Truebeam modulated the DR from 400 to 355 MU min^−1^ in order for the MLCs to run at the specified maximum speed of 2.50 cm s^−1^. That is, a reduction in the DR accompanied the reduction in the MLC leaf speed in order for the correct dose to be delivered during that interval. Since the leaves did not move at a speed that exceeded their mechanical limits, the effect of the gravity on the movement of the MLCs was therefore negligible.

The MLC leaf speed assessed under dynamic gantry conditions showed agreement with the trajectory log files data with percentage differences that ranged between 0.9% and 5.7% (Table 1). The error in the MLC leaf speed was found greater in the arc sectors of lowest DR and highest MLC leaf velocity. This may be due to the corresponding low detector signal in these sectors.

The reconstruction of DR and GS as a function of gantry angle using the proposed system has allowed independent verification of VMAT delivery components with comparison to the machine log files data. Furthermore, MLC leaf motion was measured with the additional diagonal detector arrangement in the Octa device. These tests verified the accuracy of dynamic MLC leaf performance within the manufacturer's specified limit of 2.50 cm s^−1^ for both linacs under static and dynamic gantry conditions.

The main limitations in the current prototype include the finite field size which at present allows speed evaluation of only the central MLCs. This can be rectified by increasing the area of the detector. The MLC leaf speed can only be evaluated when the dose is delivered to the detector and therefore sectors, where the beam is held, are undetectable. Further investigation on the accuracy and the behavior of the inclinometer during moments of acceleration and deceleration of the gantry, improvement on the electronics' readout to reduce the unwanted noise in the data as well as incorporating the inclinometer into the motherboard of the detector's electronics which can subsequently eliminate the equipment‐induced noise resulting from mounting the inclinometer on the linac head are intended for future work.

## 5 Conclusion

This study investigated the use of solid‐state array detectors combined with an inclinometer to perform machine‐specific VMAT QA based on a set of dynamic tests that have been recommended by the NCS CoP Report 24.^8^ The system characterized with high spatial and temporal resolution demonstrated the capability to provide independent verification of dose rate, gantry speed and MLC leaf speed with high precision in both flattened and unflattened beams and with good agreement to the machine log files.

## Conflict of Interest

No conflict of interest.
