# Corrigendum: “Quality assurance of VMAT on flattened and flattening filter free accelerators using high spatial resolution detector”

**DOI:** 10.1002/acm2.12967

**Published:** 2020-07-18

**Authors:** Fatima S. Matar, Dean Wilkinson, Jeremy Davis, Giordano Biasi, Trent Causer, Iolanda Fuduli, Owen J. Brace, Nauljun Stansook, Martin Carolan, Anatoly B. Rosenfeld, Marco Petasecca

**Affiliations:** ^1^ Centre for Medical Radiation Physics University of Wollongong Wollongong Australia; ^2^ Illawarra Cancer Care Centre Wollongong Hospital Wollongong Australia; ^3^ Illawarra Health and Medical Research Institute – IHMRI Wollongong Australia; ^4^ Department of Radiology Faculty of Medicine Mahidol University Bangkok Thailand

In the article cited above, clarifications have been made in three places as listed below.

***Introduction, lines 21‐22:***

The phrase

“examined the coordination between GS and relative dose profiles”

Has been changed to

“studied the dose control and GS”

Introduction, lines 26–27:

The phrase

“Further, the measured parameters were not evaluated as a function of gantry angle”

Has been changed to

“The relative dose and GS were evaluated as a function of gantry angle”

*Section 2C. Mutual dependence of dose rate and gantry speed, lines 1–2:*

The sentence

“The mutual dependence of DR and GS during VMAT deliveries was investigated with the use of the Customer Acceptance Plan (CAP).”

Has been changed to

“The mutual dependence of DR and GS during VMAT deliveries was investigated with the DUO system using the method Barnes et al [9] applied to the Customer Acceptance Plan (CAP).”

